# A new vector system for targeted integration and overexpression of genes in the crop pathogen *Fusarium solani*

**DOI:** 10.1186/s40694-019-0089-2

**Published:** 2019-12-11

**Authors:** Mikkel Rank Nielsen, Anna Karolina Rilana Holzwarth, Emmett Brew, Natalia Chrapkova, Samba Evelyne Kabemba Kaniki, Kenneth Kastaniegaard, Trine Sørensen, Klaus Ringsborg Westphal, Reinhard Wimmer, Teis Esben Sondergaard, Jens Laurids Sørensen

**Affiliations:** 1 Department of Chemistry and Bioscience, Aalborg University, Niels Bohrs Vej 8, 6700 Esbjerg, Denmark; 2 Department of Chemistry and Bioscience, Aalborg University, Fredrik Bajers Vej 7H, 9220 Aalborg Ø, Denmark

**Keywords:** *Fusarium*, Transformation, Heterologous expression, Fluorescence, *Agrobacterium tumefaciens*-mediated transformation, Secondary metabolites, Polyketides, Fusarubin, Bostrycoidin

## Abstract

**Background:**

Besides their ability to produce several interesting bioactive secondary metabolites, members of the *Fusarium solani* species complex comprise important pathogens of plants and humans. One of the major obstacles in understanding the biology of this species complex is the lack of efficient molecular tools for genetic manipulation.

**Results:**

To remove this obstacle we here report the development of a reliable system where the vectors are generated through yeast recombinational cloning and inserted into a specific site in *F. solani* through *Agrobacterium tumefaciens*-mediated transformation. As proof-of-concept, the enhanced yellow fluorescent protein (eYFP) was inserted in a non-coding genomic position of *F. solani* and subsequent analyses showed that the resulting transformants were fluorescent on all tested media. In addition, we cloned and overexpressed the Zn(II)_2_Cys_6_ transcriptional factor *fsr6* controlling mycelial pigmentation. A transformant displayed deep red/purple pigmentation stemming from bostrycoidin and javanicin.

**Conclusion:**

By creating streamlined plasmid construction and fungal transformation systems, we are now able to express genes in the crop pathogen *F. solani* in a reliable and fast manner. As a case study, we targeted and activated the fusarubin (*PKS3*: *fsr*) gene cluster, which is the first case study of secondary metabolites being directly associated with the responsible gene cluster in *F. solani* via targeted activation. The system provides an approach that in the future can be used by the community to understand the biochemistry and genetics of the *Fusarium solani* species complex, and is obtainable from Addgene catalog #133094.

**Graphic abstract:**

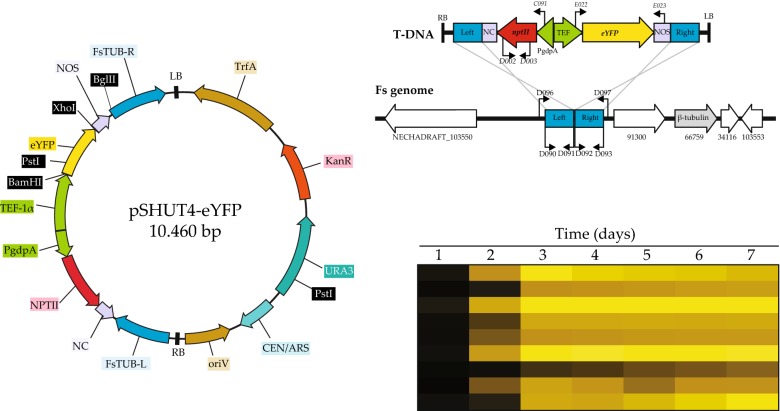

## Background

The *Fusarium solani* species complex (FSSC) is one of the most widespread fungal groups, which collectively is capable of causing disease in both plants and humans [1, 2]. More than 50 species within the FSSC have been described [1, 3, 4] and the number continuous to increase as new species are constantly identified [5], primarily based on molecular analyses. Members of the species complex are important plant pathogens of more than 100 agricultural crops where they can cause vascular wilt or root rot [6]. Individual species are often associated with only one or a few hosts and consequently plant pathogenic populations of *F*. *solani* have been further subdivided into formae speciales [7–9], although this view has been challenged recently [10]. The variation within the species complex is also reflected within sexual replication, where both heterothallic, homothallic and mitosporic species have been identified [3].

Several bioactive secondary metabolites have been identified in FSSC, including cyclosporin, gibepyrone A, lucilactaene, sansalvamide and the pigments fusarubin, javanicin and bostrycoidin [11]. Analyses of the genome of the first sequenced FSSC strain (77-13-4; FGSC 9596) revealed the presence of 13 polyketide synthases (*PKSs*) and 13 non-ribosomal peptide synthetases (*NRPSs*) [2] and subsequent orthology studies have linked fusarubin, javanicin, bostrycoidin (*PKS3*; *fsr*), gibepyrone A (*PKS8, Gpy1*), lucilactaene (*PKS10*) to their responsible gene clusters [12–17].

In the past, one of the obstacles in genetic editing of *F. solani* is the sparse reports of successful transformation protocols [18–20]. We recently overcame this obstacle through optimization of a protocol for *Agrobacterium tumefaciens*-mediated transformation (ATMT), which was used to identify the gene cluster behind biosynthesis of sansalvamide [21]. A second possible bottleneck in the transformation process can be construction of the ATMT vectors, which usually rely on correct cloning of the vector backbone containing genes for bacterial replication and selection together with two homologues recombination sequences and the integration cassette containing a selection marker and the overexpression system. Assembling these four fragments can be obtained through enzyme based methods, including Xi, *In*-*Fusion*, Gateway and USER Friendly cloning techniques [22]. The cloning process can be accelerated and made more cost-effective by in vivo homologous recombinational cloning in *Saccharomyces cerevisiae* where amplified DNA fragments are cloned guided by overlapping sequences of down to 15 bp [23, 24]. The combination of yeast based cloning and ATMT has developed for *Aspergillus fumigatus* in a time and labor reducing method [25]. The aim of the present study was to adapt this approach to *F. solani* to develop a system that allow easy generation of transformation vectors for overexpression of target genes from an active locus on the chromosome. As proof-of-concept, we chose the enhanced yellow fluorescent protein (eYFP) while the internal regulator of the *PKS3* gene cluster was targeted to demonstrate the usefulness in genetic engineering of biosynthetic gene clusters.

## Methods

### Strains, media and conditions

*Fusarium solani* mating population IV (77-13-4; FGSC 9596, Fungal Genetics Stock Center) was used as model for the transformation. The strain was maintained on Czapek Dox (Cz) agar medium or potato dextrose agar (PDA) medium [26] during the experiments. Plasmid assembly was performed in *Saccharomyces cerevisiae* BY4743 (Euroscarf Y20000, [27]), which was maintained on yeast extract peptone dextrose (YPD) medium and selected on yeast synthetic dropout medium without uracil (SC-U; Sigma-Aldrich, St. Louis, MO, USA; Y1501). *Escherichia coli* DH5α was used for yeast-plasmid recovery and propagation. Transformed cells were grown and selected on solid (2% agar) or liquid Luria–Bertani (LB, Lennox) medium supplemented with 50 µg/mL kanamycin at 37 °C. *Agrobacterium tumefaciens* AGL-1 was used for transformation of *F. solani*. The strain was grown on LB medium supplemented with 100 µg/mL rifampicin as well as 50 µg/mL kanamycin for selecting transformants carrying the assembled plasmid.

### Construction of pSHUT4-*eYFP* plasmid

**Fig. 1:**
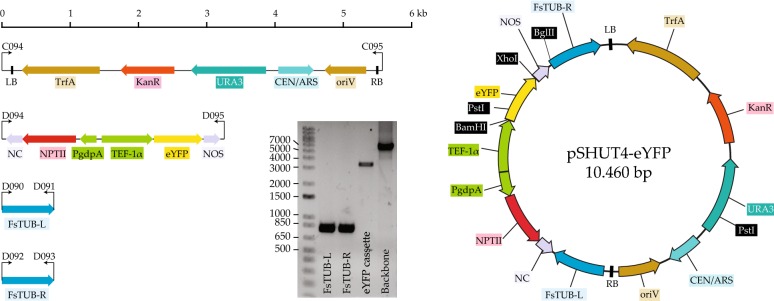
Illustration and an actual agarose of the four PCR fragments, which are subsequently assembled into pSHUT4-*eYFP* via yeast recombinational assembly

Four PCR fragments were required to generate the plasmid for constitutive expression of the *eYFP* gene from an active position on the genome. This approach was inspired from *F. graminearum*, where the aurofusarin cluster specific transcription factor *aurR1* was recently overexpressed in a locus close to the β-Tubulin gene [28]. All PCRs were performed using Phusion Hot Start II DNA Polymerase (Thermo Fisher Scientific), using the supplied reagents and manufacturer’s guidelines. Prior to the experiments, *eYFP* from the EarleyGate 104-vector [29] had been cloned into p-UGOTL-TEF1α-TF [30] to generate an expression cassette comprising the *nptII* fungal selection marker together with the constitutive translation elongation factor 1α (TEF-1α) promoter, *eYFP* and NOS terminator. This cassette was amplified using primers D094 + D095 (see Additional file 1 for all primers in this study). For guiding the homologous integration in *F. solani*, were two fragments amplified from genomic DNA using primers D090 + D091 and D092 + D093. Specifically, were the two integration border sites amplified from a non-coding region between the genes NECHADRAFT_103550 (Conserved, hypothetical *Fusarium* protein) and NECHADRAFT_91300 (Putative ATP-binding ABC transporter), found just upstream from NECHADRAFT_66759 (β-Tubulin). Finally, a plasmid backbone was PCR amplified from in-house vector pSHUT3 [31] with primers C094 + C095. This vector is based on U-GOAL [32] and comprise the bacterial elements *kan*^R^, IncP, trfA to which the yeast auxotrophic selection marker *URA3* from pYES2 (Invitrogen) and replication origin CEN6/ARSH4 from pRS315 (ATCC^®^ 77144) [33] were added. Correct assembly of the plasmid was guided by adding 15 bp homology tails to all primers yielding a 10.460 bp plasmid dubbed pSHUT4-*eYFP* (Fig. 1). The four PCR products were visualized by gel electrophoresis on 1% agarose gels and subsequently cleaned by the QIAquick PCR purification kit (Qiagen). At least 200 fmol of each of the four purified PCR fragments were transformed into *S. cerevisiae* by the lithium acetate/single-stranded carrier DNA/PEG method [34]. As controls, subsequent transformation reactions were set up where one of the four PCR fragments was omitted from the transformation mix and substituted with water. Yeast transformants were selected on solid SC-U and grown for 2 days at 30 °C. Colonies were streaked on new SC-U plates from where the plasmids were isolated from fresh yeast cells as previously described [35]. The isolated plasmids were electroporated into competent *E. coli* DH5α cells using the BioRad Micropulser Electroporation Apparatus, following the manufacturer’s instructions, and selected cells on solid LB medium with kanamycin. Assembled plasmid constructs were propagated and isolated from *E. coli* cultures using the QIAprep Spin Miniprep Kit (Qiagen). Sequencing of constructs was performed at Eurofins genomics (Ebersberg, Germany).

### Generation and transformation of *F. solani* macroconidia

Five agar plugs from a 5-day-old *F. solani* culture on Czapek Dox agar medium was added to 70 mL CMC medium [15 g/L carboxymethyl-cellulose sodium salt (C4888; Sigma-Aldrich) 1 g/L NH_4_NO_3_, 1 g/L KH_2_PO_4_, 0.5 g/L MgSO_4_∙7H_2_O, 1 g/L Bacto yeast extract (Becton, Dickinson and Company, Sparks, MD, USA] [36] in a 250 mL baffled flask. The culture was incubated in the dark for 5 days at 20 °C with 100 rpm. The medium was filtered through a sterilized syringe lightly packed with glass wool before the flow through was centrifuged at 5000 g for 40 min at 5 °C. The supernatant was carefully discarded and the pellet resuspended in 10 mL cold sterile H_2_O. This step was repeated twice with centrifugation for 20 min, before the macroconidia were ready for transformation.

The *F. solani* macroconidia were transformed with pSHUT4-*eYFP* using *A. tumefaciens* AGL1 as previously described [21]. The transformation mixture containing *A. tumefaciens* and *F. solani* was spread onto induction medium plates containing a black filter paper (Frisenette, Denmark, 140.090). Positive controls included plates with and without filters plated with either plasmid carrying *A. tumefaciens* or macroconidia. After incubation at 22 °C for 3 days, the filters were moved to V8 agar plates [37] containing 150 µg/mL G418 (Gibco) and 300 µg/mL cefoxitin sodium (Sigma-Aldrich). After 7 days, the filters were moved to new V8 agar plates containing 150 µg/mL G418 where colonies started to emerge after 1–5 days. Single colonies were transferred to potato dextrose agar (PDA) [26] plates containing 200 µg/mL G418. All incubation steps were carried out in darkness.

### Verification of transformants by PCR and sequencing

Colony PCR was routinely used to screen isolated mutants with primers hybridizing outside integration locus and inside the T-DNA cassette. A minute amount of 2–3 day old hyphal mycelium was picked with a sterile toothpick and submerged in 300µL fungal lysis buffer [0.2 M NaCl, 0.1% Triton-X100, 0.2% SDS, 10 mM Tris–HCl, 50 mM EDTA, pH 7.5] and vortexed vigorously for 1 min before spinning at 10,000×*g* for 3 min. 1µL supernatant can be applied in standard PCR mix with the addition of 2 mg/mL bovine serum albumin (Sigma-Aldrich; A9418). Genomic DNA of selected *F. solani* transformants was isolated from 2 day old mycelium grown in YPG cultures at 25 °C shaking 150 rpm using the DNeasy plant mini kit (Qiagen) and a series of PCR reactions were used to verify that the cassette had been integrated correctly into the genome.

High-molecular weight genomic DNA was extracted from 50 mg freeze-dried mycelium grown in 100 mL YPG-medium in 2 days at 25 °C. Extraction was preformed using DNeasy powersoil kit (QIAGEN), with following modifications: samples were homogenized for 1 min on a vortex adaptor instead of 10 min and all “vortex to mix” steps were excluded, and mixing was done by invert the tubes instead. The high molecular weight genomic DNA was prepared for sequencing using the 1D Native barcoding genomic DNA kit (EXP-NBD104 and SQK-LSK109) (Oxford Nanopore Technologies, Oxford, UK) and the sequencing was performed on a FLO-MINI106D (R9.4) flow-cell (Oxford Nanopore Technologies, Oxford, UK) with approximately 40–300× coverage. Base-calling was preformed using Guppy v. 2.1.3 and demultiplexing and adaptor trimming was preformed using Porechop v. 0.2.3. The adaptor trimmed reads was filtered using Filtlong, reads less than 10.000 bp and with a mean quality score under 80 were excluded. Mapping of reads to the corresponding mutant reference genome was preformed using Minimap2.

### Quantification of fluorescence in the OE::*eYFP* transformant

One of the verified transformants (OE::*eYFP*-6) was subjected to functional analyses. The strain was grown on Cz agar together with the wild type and analyzed via microscope. Pictures were taken at 10X magnification on an Olympus IX83 confocal quipped with a Yokogawa CSU-W1 spinning disk and a Hamamatsu ORCA-Flash 4.0 camera. EFI projections were made from Z-stacks with 1.99 um Z-spacing (20- 48 frames) at 508 nm emission and 489 nm excitation wavelengths. Furthermore, The strain was grown together with the wild type at 25 °C in 24 well polypropylene plates containing 1 mL of yeast mold (YM) medium, malt extract (ME) medium, potato dextrose (PD) medium, yeast extract sucrose (YES) medium, YPD medium, (Cz) medium, Cz with yeast extract (CzY), Cz with 1 M NaCl and Cz with NaNO_3_ replaced with (NH_4_)_2_SO_4_ [26]. Growth and fluorescence of both strains in four replicates were measured daily using an Infinite m1000pro (Tecan, Männeforf, Switzerland). Optical density (600 nm) was used to quantify growth and excitation/emission wavelength of 514/526 was used to quantify eYFP.

### Generation of a *fsr6* overexpression *F. solani* transformant

Identification of the *fsr6* gene was done by BLASTx comparisons of known *Fusarium* fusarubin (PKS3) cluster transcription factor sequences *F. fujikuroi fsr6* (FFUJ_03989, [38]) and *F. graminearum pglR* (FGSG_09188, [39]) towards the *F. solani* genome. We identified a 1167 bp gene, positions 251,539–252,705 on chromosome 10 (Additional file 2), which was recognized as an open reading frame on the reverse strand by GENSCAN [40] with the predicted function of a Zn(II)_2_Cys_6_ fungal transcription factor. To overexpress the fusarubin gene cluster in *F. solani* we chose a strategy were *eYFP* was substituted by the internal regulator *fsr6*. Initially, *eYFP* was removed from the pSHUT4-*eYFP* vector using *Bam*HI and *Xho*I digestion. The digest was run on a 1% agarose gel and the linearized pSHUT4 backbone was purified with the QIAquick Gel Extraction Kit (Qiagen). The *fsr6* gene was amplified by PCR using the primers D100 + E001, which each contained 20 bp overlaps to the linearized pSHUT4 backbone. The *fsr6* gene and the pSHUT4 backbone were assembled in *S. cerevisiae* yeast recombinational assembly as described above. The verified pSHUT4-*fsr6* vector was transformed into *F. solani* macroconidia as described above. The transformation yielded a single transformant, which was verified by colony PCR, short-read sequencing (Additional file 6) and whole genome sequencing as described above.

### Determination of growth and pigment production in the OE::*fsr6* transformant

The radial growth of the OE::*fsr6* mutant was initially measured and compared to the parental *F. solani* wild type strain. The experiment was performed on solid Cz and YES medium in 90 mm petri dishes, which were inoculated with 1000 spores (resuspended in 0.2% agar) at the center of each plate using three replicates of each strain. The plates were incubated at 28 °C with daily growth measurements for 5 days where a cardinal axis was drawn from the inoculation point to facilitate the measurements in X and Y radii lengths. The colony area was estimated as Area = r_X_∙r_Y_∙π.

To compare pigment production in the OE::*fsr6* strain to the wild type *F. solani* strain, 5000 spores were added to 50 mL YES medium in 250 mL baffled flasks in triplicates. The flasks were incubated for 6 days at 28 °C and 100 rpm. The produced pigments in the broth was extracted using a mixture of chloroform and methanol as previously described [28]. The chloroform phase was evaporated to dryness on a rotary evaporator at 40 °C and resuspended in 1 mL DMSO. The extracts were analyzed on a Hitachi Elite LaChrom HPLC system equipped with a 150 × 4.6 mm Ascentis Xpress 2.7 mm phenyl-hexyl column (Sigma Aldrich, USA) and coupled to a high resolution mass spectrometer (compact qTOF, Bruker, Germany) operating in positive ionization mode using the settings as previously described [41].

To verify the identity of bostrycoidin and javanicin, twenty 0.6 mm plugs from the OE::*fsr6* transformant were used to inoculate 2 × 1 L YES medium. The culture was grown for 7 days at 25 °C and 105 rpm, before the medium was extracted as described above. The resulting extract was subjected to purification by a preparative HPLC (Waters HPLC pump 515; Waters Binary Gradient Module 2545; Waters System Fluidics Organizer) coupled to a quadrupole mass analyzer (Waters Acquity QDa), a UV/Vis detector (Waters UV/Visible detector 2489) and a fraction collector (Waters Sample Manager 2767). The sample was separated by a C6-phenyl column (Phenomenex Gemini 5 µm C6-Phenyl, 110 Å, 250 × 10 mm) by injecting 1 mL per run and applying a linear gradient of water (VWR, HiPerSolv CHROMANORM HPLC–MS grade) and acetonitrile (VWR, HiPerSolv CHROMANORM HPLC–MS grade), both supplemented with 0.1% formic acid (VWR, 99% HiPerSolv CHROMANORM LC–MS grade). The gradient initiated at 10% acetonitrile, increased to 99% over 15 min, and held at 99% for 5 min with a constant flow at 10 mL/min. The system was controlled by MassLynx V4.2 set to collect the masses M = 290.1 Da and M = 285.1 Da. Javanicin and bostrycoidin eluted after 9.3 and 10.0 min, respectively. The collected fractions were lyophilized and resuspended in CDCl_3_ (euroiso-top, 0.03% tetramethylsilane (TMS)) and ^1^H nuclear magnetic spectroscopy (NMR) spectra were recorded on a Bruker AVIII-600 MHz spectrometer (Bruker, Karlsruhe, Germany) equipped with a triple resonance cryogenically cooled probe with z-gradients and controlled by TopSpin 3.5pl6. All spectra were recorded at 298.1 K and calibrated to internal TMS. The obtained spectra were compared to published data for javanicin and bostrycoidin [42, 43].

## Results and discussion

### Assembly of shuttle vector pSHUT4::*eYFP* by yeast mediated recombinational cloning

To generate pSHUT4-*eYFP* four PCRs were performed to amplify the backbone, expression cassette and the two integration border sites using primers carrying 15 bp homology to the neighboring fragment (30 bp total homology between all fragment pairs). The resulting products were visualized by gel electrophoresis, which matched the expected sizes (Fig. 1). The four fragments were transformed into *S. cerevisiae* and six resulting colonies were selected to verify that the plasmid had been correctly assembled. After propagation in *E. coli,* the plasmids were digested with PstI and BglII, which resulted in the anticipated band pattern. Three plasmids were validated further by PCR yielding identical and correct band lengths. The *eYFP* cassette was ultimately sequenced in one of the plasmids, which confirmed that the plasmid had been correctly assembled (Additional file 3).

### Fungal transformation and validation

**Fig. 2:**
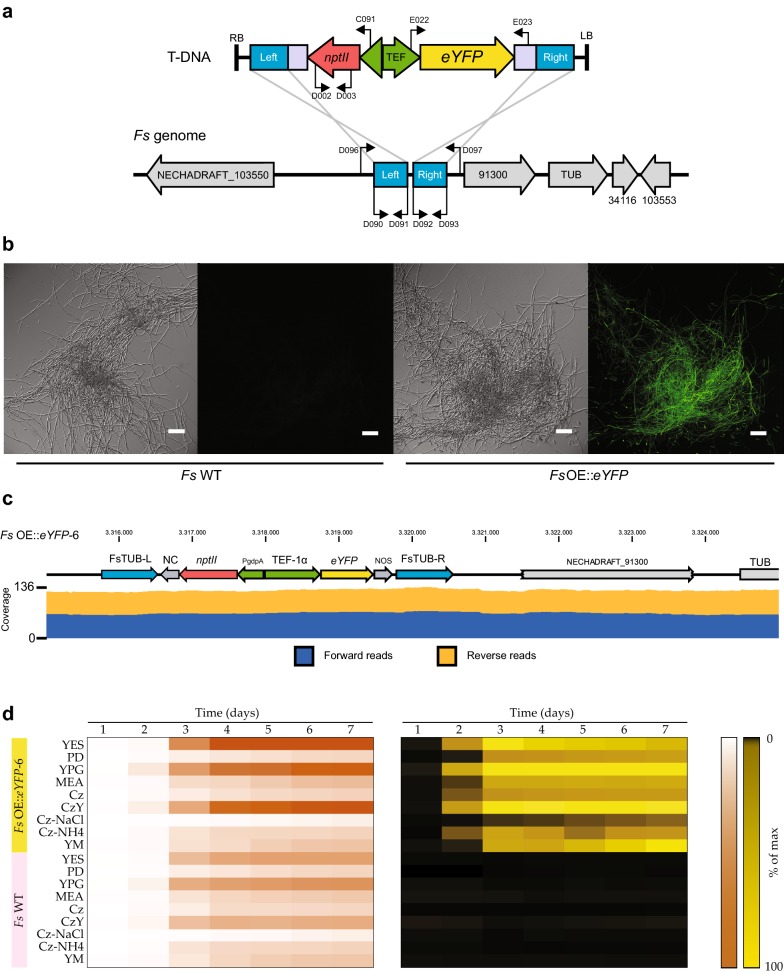
Construction and validation of *eYFP* overexpression mutant *Fs* OE::*eYFP*-6. **a** Integration of pSHUT4-*eYFP* T-DNA cassette into a non-coding position of the *F. solani* genome through homologous recombination between identical segments (blue). Primer positions are marked, and a full list of primers is found in Additional file 1. **b** DIC pictures and EFI projections comparing parental and mutant strain phenotypes. Scale bar = 100 µm. **c** Genome sequencing validation displays correct integration of the pSHUT4-*eYFP* T-DNA cassette via homologous recombination. **d** Growth and *eYFP* induction of *F. solani* wild type and OE::*eYFP*-6 during growth on nine different media. The left panel illustrates growth (optical density) over a 7 days period. The right panel illustrates fluorescence at excitation/emission wavelength of 514/526. All data points are the average of four biological replicates, expressed as a percentage of the maximum observed value

The verified pSHUT4:*eYFP* was transformed into *F. solani* by ATMT, which resulted in more than 30 fungal colonies from 10 plates, equal to 25 fungal colonies per 10^7^ conidia. Seven randomly picked colonies were streaked on selective PDA of which six showed uninhibited growth. Subsequent diagnostic colony PCR showed furthermore that the T-DNA had correctly recombined into the integration site in all colonies tested (Primer positions marked in Fig. 2a). Two of the transformants displaying fluorescence (Fig. 2b), OE::*eYFP*-2 and -6, were selected for further validation using a PCR based strategy. The initial PCR confirmed that the transformants contain the *nptII* gene. The following PCR with primers D096 + D097 yielded only a product in the wild type, possibly because the theoretical size of 5983 bp in the transformants was too large for successful amplification. This suggests that the T-DNA had been inserted into the correct locus, which was further verified when primers targeting the T-DNA were combined in PCRs with primers targeting the up- and downstream regions (Additional file 4). Following a short-read sequencing of the final two PCR products validating correct recombination, we performed whole genome sequencing of the mutant ultimately verifying correct integration (Fig. 2c).

### Functional characterization of *F. solani eYFP* transformants

To determine that *eYFP* was functionally expressed by the TEF-1α promoter from the integration site, OE::*eYFP*-6 was selected for further analyses in a time study together with the wild type. The results showed that fluorescence could be detected after 2 days on all media, except for PDA and Cz-NaCl, which was delayed by 1 day (Fig. 2d). The fluorescence signal continued throughout the experiment on all media, although a decrease followed by stagnation was observed on YES, YPG and Cz. This could indicate the cultures had reached a phase with reduced cellular activity caused by nutrient depletion. The observation that the promoter is active on all the media tested is valuable information in future studies where the examined target genes require a specific growth medium or condition.

### Targeted activation of the *PKS3* gene cluster results in production of javanicin and bostrycoidin pigments

**Fig. 3:**
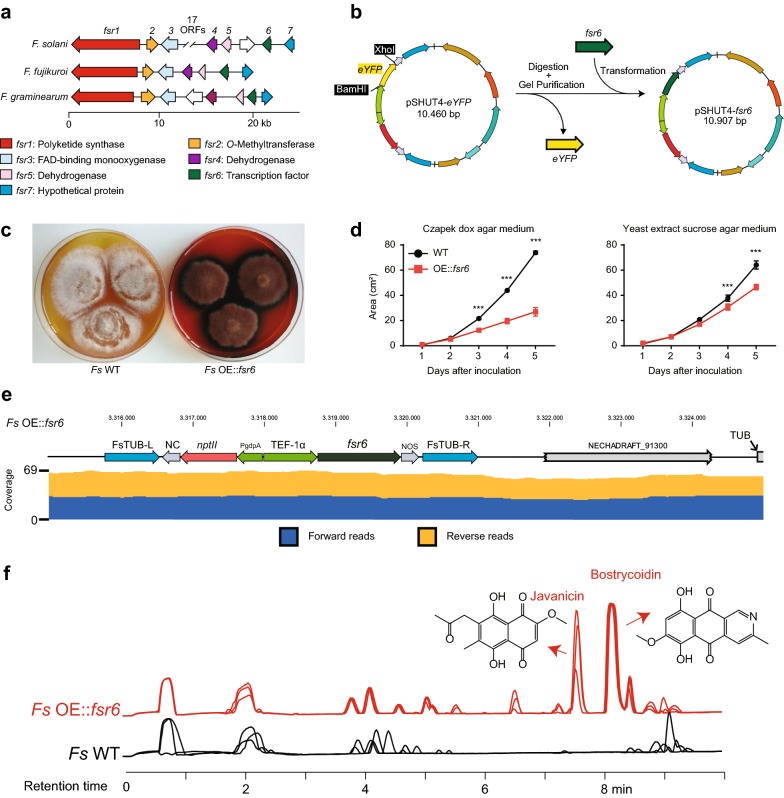
Construction and validation of overexpression mutant *Fs* OE::*fsr6*. **a** Illustration of *fsr* gene cluster orthologues in *Fusarium solani*, *F. fujikuroi* and *F. graminearum*. **b** Workflow for replacement of the *eYFP* gene for *fsr6* in the agrobacterium shuttle vector pSHUT4 via yeast mediated recombinational assembly. **c** Photographs of *F. solani* wild type and *fsr6* overexpression mutant. **d** growth diameter test on Czapek dox and yeast sucrose agar media. **e** genome sequencing of *Fs* OE::*fsr6* reveals correct integration of the pSHUT4-*fsr6* T-DNA cassette in the locus via homologous recombination. **f** HPLC–MS total ion chromatograms for metabolite extracts from *F. solani* wild type and the *fsr6* overexpression mutant in triplicates. The compounds javanicin [M + H]^+^ = 291.0868 and bostrycoidin [M + H]^+^ = 286.0715 were observed in the mutant extracts

We identified an orthologue to the local transcription factor *fsr6* controlling activation of the *PKS3* biosynthetic gene cluster in *F. fujikuroi* (Fig. 3a). The gene was rapidly replaced in the *Agrobacterium* expression cassette of pSHUT4-*eYFP* yielding the plasmid pSHUT4-*fsr6* (for plasmid validation, see Additional file 5) (Fig. 3b). The fungal transformation yielded a single mutant with a distinct deep red/purple phenotype, which is visibly different from the white color of the wild type when grown on PDA medium (Fig. 3c). In addition to the *fsr6* gene, we applied the presented system to introduce and express several other genes in *F. solani* (not shown). In our experience, the system, on average, yields one transformant per plate. During the isolation process of the OE::*fsr6* transformant, we observed a reduced growth rate. The transformant was therefore grown on Cz and YES agar medium together with the wild type. This experiment confirmed that the transformant grew significantly slower than wild type (Fig. 3d). This reduced growth rate could be due to the accumulation of pigments and thereby be responsible for the low transformation success. The transformant was subsequently screened by diagnostic PCR, which indicated that the expression cassette had been correctly inserted (Additional file 6). This was further validated by whole genome sequencing of *Fs* OE::*fsr6* (Fig. 3e). Analysis on HPLC-HRMS of the secondary metabolites produced in liquid cultures of and wild type *F. solani* strain and the OE::*fsr6* displayed two dominant peaks not visible in wild type extracts at 7.5 and 8.2 min with masses matching javanicin and bostrycoidin (Fig. 3f). These compounds were subsequently isolated by preparative HPLC and their identity confirmed by NMR (Additional files 7 and 8). These two compounds are derived from the *PKS3* cluster and thereby confirming functional integration of the *fsr6* overexpression cassette into *F. solani*.

### Future potential of the vector system

The aim of our study was to develop a platform for reliable targeted transformation of *F. solani*, which can be used to increase our understanding on this important pathogenic fungus. One research area that can benefit from the platform is the secondary metabolite research in *Fusarium*. So far, the only secondary metabolite to be linked to a gene directly in *F. solani* is sansalvamide [21]. However, comparative analyses have revealed that *F. solani* contain numerous biosynthetic gene clusters, including 13 polyketide synthases (*PKSs*) of which only three can be linked to a product based on orthology [12–14, 16, 17, 44]. The pSHUT4-*eYFP* vector can be modified and used to overexpress local transcription factors of biosynthetic gene clusters, which has proven a highly successful approach to identify novel secondary metabolites [45]. This approach has already been adapted in other *Fusarium* species, which for example has led to discovery of fusarielins in *F. graminearum* [46], equisetin in *F. heterosporum* [47] and fujikurins in *F. fujikuroi* [48]. The simple workflow needed to overexpress a target gene (e.g. transcription factor) is illustrated in Fig. 2b. First, the *eYFP* has to be looped out by digesting the plasmid with XhoI and BamHI. The target gene can then be inserted into the linearized plasmid by yeast recombinational cloning and transferred into an active position in the *F. solani* genome by ATMT.

Among the 13 *PKSs* in *F. solani*, we have identified putative transcription factors for seven gene clusters for which we have adopted this system. Other fungal species can however also be targeted in a similar approach, the only requirement is that guiding integration border sites should be replaced with a suiting region from the target organism. Potentially, the presented vector could be used to transform closely related members of the FSSC given the integration locus shares high sequence similarity to the integration site in *F. solani* f. sp. *pisi*. A similar locus in the genome of *F. neocosmosporiellum* shares 86% nucleotide content with the integration border sites [49], however, whether pSHUT4 can successfully be applied in such a transformation experiment remains to be tested. It should be noted by researchers wishing to apply the system for genetic studies concerning signal transduction or virulence etc., we recommend carefully monitoring potential change the expression of neighboring genes when introducing any expression cassette, as introduction of recombinant DNA may compromise the intrinsic regulation. The system is not restricted to ATMT as linearized or circular plasmids can be used for protoplast transformation in microorganisms where this is feasible.

## Conclusions

In this study we present the implementation of a novel vector system that allows for fungal transformation and strong gene expression in the crop-pathogen *F. solani*. As a case study, we performed targeted activation of the biosynthetic *fsr* gene cluster responsible for mycelial pigmentation. From cultivation experiments in this study, we were able to isolate and confirm the chemical structures of the red pigments javanicin and bostrycoidin. Targeted overexpression of transcriptional regulators is thus a feasible approach to activate biosynthetic gene clusters as a venue for isolating and describing novel secondary metabolites in the FSSC.

## Supplementary information

**Additional file 1.** List of primers.

**Additional file 2.** *fsr6* nucleotide sequence.

**Additional file 3.** pSHUT4-*eYFP* plasmid validation.

**Additional file 4.** *F. solani* OE::*eYFP* mutant colony PCR validation.

**Additional file 5.** pSHUT4-*fsr6* plasmid validation.

**Additional file 6.** *F. solani* OE::*fsr6* mutant validation by PCR and TubeSeq sequencing.

**Additional file 7.** NMR table of javanicin isolated from *Fs* OE::*fsr6*.

**Additional file 8.** NMR table of bostrycoidin isolated from *Fs* OE::*fsr6.*

## Data Availability

The whole genome sequencing datasets analyzed during the current study are available from the corresponding author on reasonable request. The sequences of primers included in this article can be found in Additional file 1. The pSHUT4-*eYFP* plasmid is available at the Addgene repository (# 133094).

## Abbreviations

ATMT: *Agrobacterium tumefaciens*-mediated transformation
CMC: carboxymethyl-cellulose
Cz: Czapek Dox
CzY: Czapek Dox-Yeast extract
DIC: differential interference contrast microscopy
EFI: extended focal image
eYFP: enhanced yellow fluorescent protein
Fs: *Fusarium solani*
FSSC: *Fusarium solani* species complex
HPLC-HRMS: high-pressure liquid chromatography-High resolution mass spectrometry
ME: malt extract
OE: overexpression
PCR: polymerase chain reaction
PD: potato dextrose
PKS: polyketide synthase
qTOF: quadropole time-of-flight
SC-U: yeast synthetic dropout medium without uracil
T-DNA: transfer-DNA
YES: yeast extract-sucrose
YM: yeast mold
YPG: yeast extract-peptone-glucose
