# Supplementary material for: A new vector system for targeted integration and overexpression of genes in the crop pathogen Fusarium solani
Source: Fungal Biol Biotechnol. 2019 Dec 11;6:25. doi: 10.1186/s40694-019-0089-2 (PMC6905090; doi:10.1186/s40694-019-0089-2)
Supplement: Supplementary file 1 — Additional file 1. List of primers. [file 40694_2019_89_MOESM1_ESM.pdf]

Supplementary data for

"A new vector system for ectopic gene expression in the crop pathogen Fusarium solani"

by Nielsen MR, Holzwarth AKR, Brew E, Chrapkova N, Kaniki SEB, Kastaniegaard K, Sørensen T, Westphal KR,

Wimmer R, Sondergaard TE and Sørensen JL.

Additional file 1: List of primers

Table 1. Primers used in this study.

Number	Sequence[1]	Usage
C094	**GGAATTCGTGGCATC**AACGTCGTGACTGGGAAAAC	Vector backbone
C095	**CTTATGGACTTGGTG**CGCTCATGATCAGATTGTCG	
D090	**ATCTGATCATGAGCG**CACCAAGTCCATAAGCCACA	Locus left boarder
D091	**ATAACGACTTTGGCC**CCATGAGCCTCGAAATAGGA	
D092	**AGTGTATTAATTTCG**TCCTGAAAAGGTCCACATGA	Locus right boarder
D093	**CCCAGTCACGACGTT**GATGCCACGAATTCCAATCT	
D094	**TTTCGAGGCTCATGG**GGCCAAAGTCGTTATTAATGC	YFP expression cassette
D095	**TGGACCTTTTCAGGA**CGAAATTAATACACTGACAGAGACG	
D096	AGGCAAAGCAAGGTAAAGCA	Validation
D097	GCAGTTTCTCCACAGCCTTC	
E022	TTTCCCCCATACCTCCTTTC	
E023	AAGACCGGCAACAGGATTC	
D002	GTAAAGCACGAGGAAGCG	
D003	TTGGGTGGAGAGGCTATT	
C091	CTGCCTCTTACCACCTGCTC	
D005	AGCATGGTGAACCACACAAA	
E086	GTGACAGGGTCACCCTGGGA	
D100	**AGTTCGCACCACCTTCAAAG**ATGACCATGCTATCTGATCCGT	*fsr6*
E001	**CAAATGTTTGAACGATCGGC**TTACGCCCATTCTCCCTGA	

[1] Nucleotides in bold create overlaps to neighboring fragments for homologous recombination during yeast recombinational assembly.
