# Supplementary material for: A new vector system for targeted integration and overexpression of genes in the crop pathogen Fusarium solani
Source: Fungal Biol Biotechnol. 2019 Dec 11;6:25. doi: 10.1186/s40694-019-0089-2 (PMC6905090; doi:10.1186/s40694-019-0089-2)
Supplement: Supplementary file 2 — Additional file 2. fsr6 nucleotide sequence. [file 40694_2019_89_MOESM2_ESM.pdf]

Supplementary data for

"A new vector system for ectopic gene expression in the crop pathogen Fusarium solani"

by Nielsen MR, Holzwarth AKR, Brew E, Chrapkova N, Kaniki SEB, Kastaniegaard K, Sørensen T, Westphal KR, Wimmer R, Sondergaard TE and Sørensen JL.

Additional file 2: *fsr6* nucleotide sequence

>GG698970.1(complement[251539..252705])_fsr6
ATGACCATGCTATCTGATCCGTTTCGAGTTATCGACGACCATGCGTCCACCATCACACCACCTGTGCCGCGTCCACAGAAACCTGTTCGA
CTGAGACTCGCTTGCGATGCCTGCACTGCGGCCAAGGTTAGGTGTAGCAAGACTCACCCTTGTGAGCGGTGCATAGACAATGGGCAGG
AGTGCTTTTACAGCGCTTCTCGACGACATGGCAAGAGATCTCGACATAGAAAGACCGAGTCTGATGCTCAACGCTCCTCGTCAAGCGAG
ATACCAACGCCTGGAGCTATTCCAGTGCCAAACGGGATGAAGGGCATGTCTTATGGCTCCGAGTTCTCTTGGGAAGAGAGGGAGACAA
GCCCACAGTTGTCAAGCTACCAAGCATCCACTCTGGGCAACGGTATCGGAAAACTCGACAACTGGGCCTTCCACGACATTGACATGATG
TTCGACTTTGACGACACGTCCTATATATCCCTAGCCGAACCATGGAAAACAACACCCGGATCCCTCGCGAGCCAACAAACCCTCAGCGA
AACAATCACCCCTCCCGAACAGCACACAACACCTGCAGAGCAACCAATCAATACTACCTCGACGCCGCCGATCGAGCCCCACGATCCCA
AACAAGCACACGACTGCGAGGCTCTAGCTCTTGGCGTTCTCCGCTCGCTGCATCACCACAACGCAGATCGCATCTGCAAGGCACCTGCA
GCAGCCAGCGGCATGAACCTCGCCAAGCCGATGCCAAGCATCGACACGGTCCTGTTTGCCAACAAGGCTGCCCTGACCAATCTCATCCC
CCTGCTCAAGTGTCCATGTGCGAGGAACCCGCATATTGCTCTTTTGCATAGCACCATTCTTTCCAAGACCATCTTTTGGTACCGTGTTGCT
GTTACTGCGCGGTATCATGCCGAGGGAGCGGAATTGCGGCCGATGAAGATACAGTTGGGGATGCTGGATCTTGACGATGATGATCAG
GCTACTTTGCAGCGAGCTGTGCTTCTTAGGGAGCTACGCAAGGCGGAAAAGGTCATGGAGACGTTTGATGAGTGTTCTGCCAGTGAGG
ATGAGGTGCCAGAGTGGCATGCTACGGCTATTCAGAACATGAAGGAGGAGCTTCAGGCGATTATTCAGAAGATCAAGAAGGGTCAGG
GAGAATGGGCGTAA
