# Supplementary material for: A new vector system for targeted integration and overexpression of genes in the crop pathogen Fusarium solani
Source: Fungal Biol Biotechnol. 2019 Dec 11;6:25. doi: 10.1186/s40694-019-0089-2 (PMC6905090; doi:10.1186/s40694-019-0089-2)
Supplement: Supplementary file 3 — Additional file 3. pSHUT4-eYFP plasmid validation. [file 40694_2019_89_MOESM3_ESM.pdf]

Supplementary data for

"A new vector system for ectopic gene expression in the crop pathogen Fusarium solani"

by Nielsen MR, Holzwarth AKR, Brew E, Chrapkova N, Kaniki SEB, Kastaniegaard K, Sørensen T, Westphal KR,

Wimmer R, Sondergaard TE and Sørensen JL.

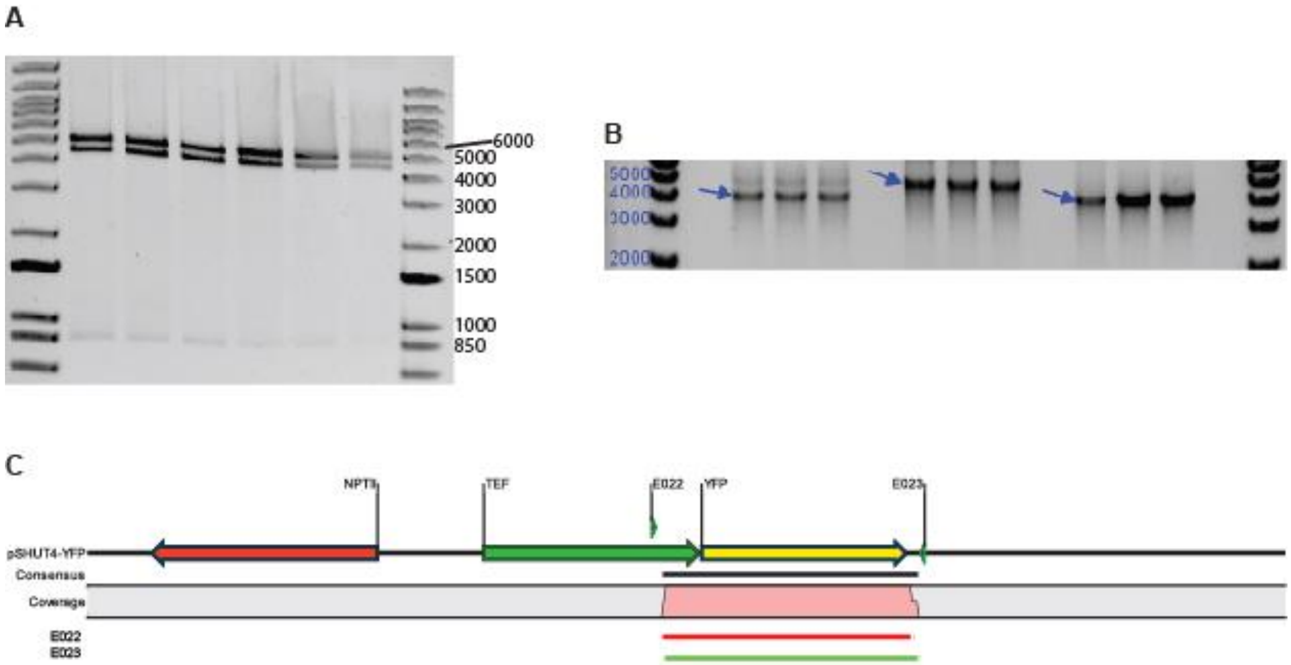

Additional file 3: pSHUT4-*eYFP* plasmid validation. A. PstI and BglII digests (5094 + 4515 + 863 bp) of 6 isolated yeast assembly clone minipreps. Restriction enzyme recognition sites are labelled in **Figure 1**. B. PCR verification was performed on three selected minipreps with primers annealing as indicated in **Figure 1**: D090+D095; 4050 bp, D090+D093; 4832 bp, D094+D093; 4059 bp. C. TubeSeq sequencing of a single selected construct was performed at Eurofins genomics (Ebersberg, Germany) using primers E022 and E023.
