# Supplementary material for: A new vector system for targeted integration and overexpression of genes in the crop pathogen Fusarium solani
Source: Fungal Biol Biotechnol. 2019 Dec 11;6:25. doi: 10.1186/s40694-019-0089-2 (PMC6905090; doi:10.1186/s40694-019-0089-2)
Supplement: Supplementary file 4 — Additional file 4. F. solani OE::eYFP mutant colony PCR validation. [file 40694_2019_89_MOESM4_ESM.pdf]

Supplementary data for

"A new vector system for ectopic gene expression in the crop pathogen Fusarium solani"

by Nielsen MR, Holzwarth AKR, Brew E, Chrapkova N, Kaniki SEB, Kastaniegaard K, Sørensen T, Westphal KR, Wimmer R, Sondergaard TE and Sørensen JL.

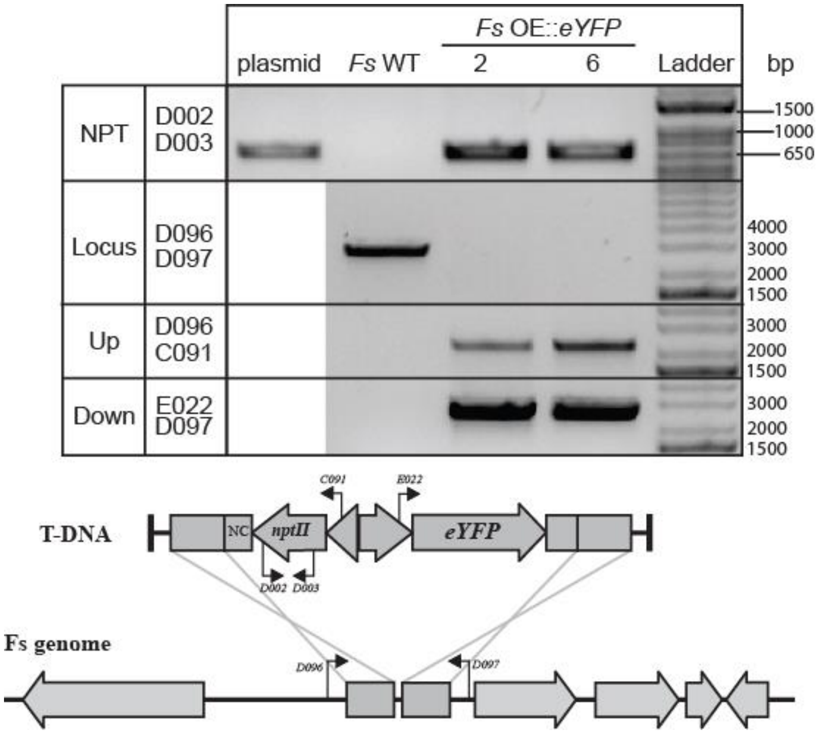

Additional file 4: *Fs* OE::*eYFP* mutant colony PCR validation. As an initial screen, two randomly selected mutants overexpressing the *eYFP* gene was analyzed with colony PCR. The expected product lengths are: NPT; 691 bp. Locus; 2715 bp for the unmodified locus, and 5536 bp for a locus containing the insert, which we assume exceeds the upper limitations of a colony PCR performed at the described reaction conditions. Up; 2206 bp. Down; 2490 bp.
