# Supplementary material for: A new vector system for targeted integration and overexpression of genes in the crop pathogen Fusarium solani
Source: Fungal Biol Biotechnol. 2019 Dec 11;6:25. doi: 10.1186/s40694-019-0089-2 (PMC6905090; doi:10.1186/s40694-019-0089-2)
Supplement: Supplementary file 5 — Additional file 5. pSHUT4-fsr6 plasmid validation. [file 40694_2019_89_MOESM5_ESM.pdf]

Supplementary data for

"A new vector system for ectopic gene expression in the crop pathogen Fusarium solani"

by Nielsen MR, Holzwarth AKR, Brew E, Chrapkova N, Kaniki SEB, Kastaniegaard K, Sørensen T, Westphal KR,

Wimmer R, Sondergaard TE and Sørensen JL.

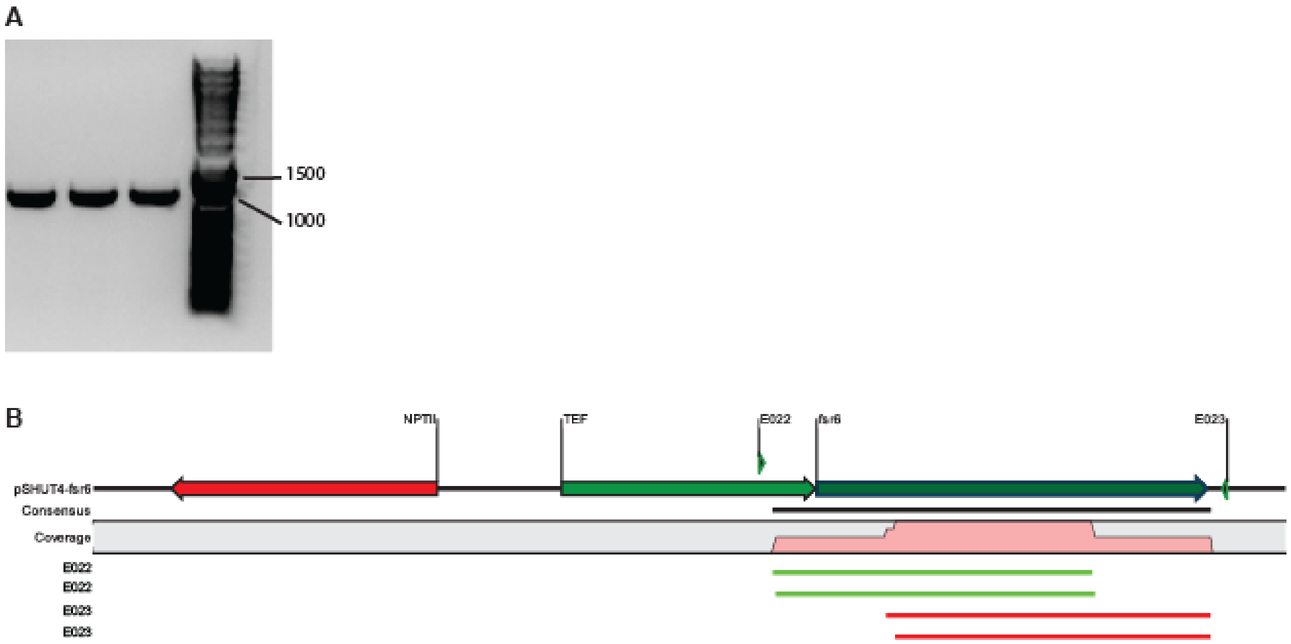

Additional file 5: pSHUT4-*fsr6* plasmid validation. A. Primers E022+E023; 1399 bp annealing outside the insertion site (**Figure 2**) were used to validate three plasmid clones. B. Two plasmid clones were sequenced at Eurofins genomics (Ebersberg, Germany) using primers E022 and E023.
