# Supplementary material for: A new vector system for targeted integration and overexpression of genes in the crop pathogen Fusarium solani
Source: Fungal Biol Biotechnol. 2019 Dec 11;6:25. doi: 10.1186/s40694-019-0089-2 (PMC6905090; doi:10.1186/s40694-019-0089-2)
Supplement: Supplementary file 6 — Additional file 6. F. solani OE::fsr6 mutant validation by PCR and TubeSeq sequencing. [file 40694_2019_89_MOESM6_ESM.pdf]

Supplementary data for

"A new vector system for ectopic gene expression in the crop pathogen Fusarium solani"

by Nielsen MR, Holzwarth AKR, Brew E, Chrapkova N, Kaniki SEB, Kastaniegaard K, Sørensen T, Westphal KR,

Wimmer R, Sondergaard TE and Sørensen JL.

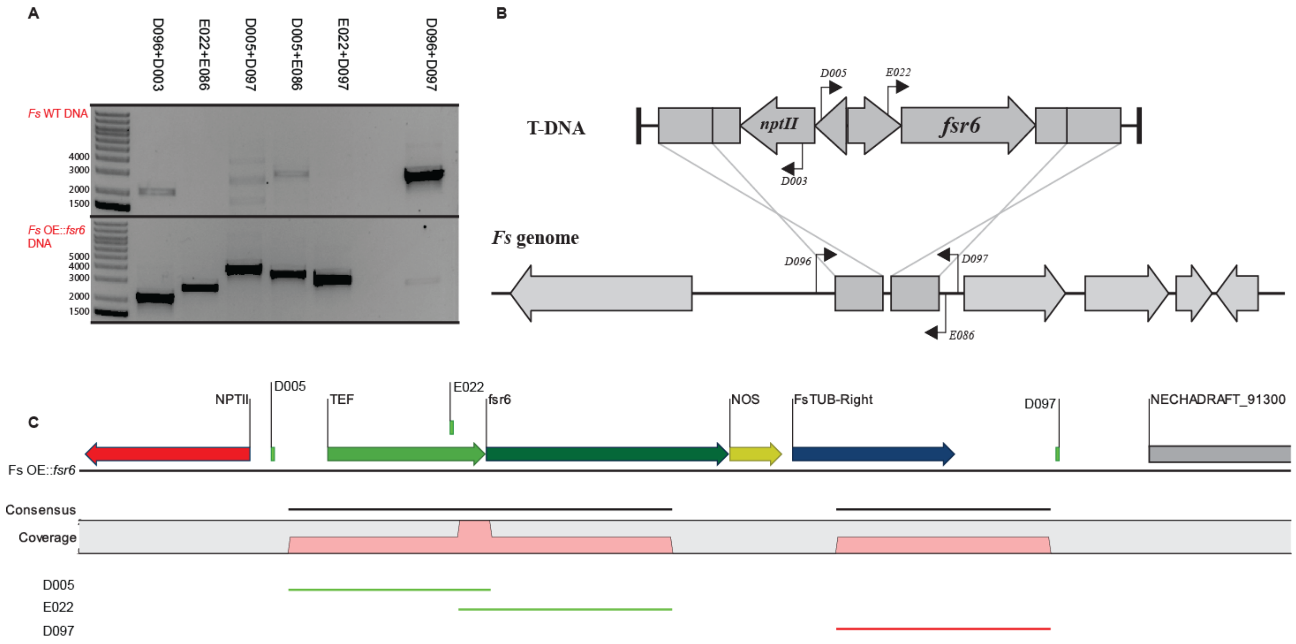

Additional file 6: *Fs* OE::*fsr6* mutant validation by PCR and TubeSeq sequencing. A, Top: PCR reactions on F. solani parental strain genomic DNA. A, below: PCR products displaying presence of intact cassette inside the targeted locus in the mutant. B. Primer positions. C. Sequencing reads display presence of expression cassette and targeted homologous recombination. All primers are listed in **Additional file 1**.
