# Supplementary material for: A new vector system for targeted integration and overexpression of genes in the crop pathogen Fusarium solani
Source: Fungal Biol Biotechnol. 2019 Dec 11;6:25. doi: 10.1186/s40694-019-0089-2 (PMC6905090; doi:10.1186/s40694-019-0089-2)

Supplementary data for

"A new vector system for ectopic gene expression in the crop pathogen Fusarium solani"

by Nielsen MR, Holzwarth AKR, Brew E, Chrapkova N, Kaniki SEB, Kastaniegaard K, Sørensen T, Westphal KR,

Wimmer R, Sondergaard TE and Sørensen JL.

Additional file 7: NMR table of javanicin isolated from *Fs* OE::*fsr6*

Signal	Javanicin ppm (Chowdhury *et at*, 2017)[1]	ppm (obs)	ΔPPM
1	13.25 (1H, s, -OH)	13.24	0.01
2	12.85 (1H, s, -OH)	12.85	0.00
3	6.20 (1H, s, -CH)	6.20	0.00
4	4.02 (3H, s, $-OCH_3$)	4.02	0.00
5	3.92 (2H, s, $-CH_2$)	3.90	0.02
6	2.32 (3H, s, $-CH_3$)	2.30	0.02
7	2.28 (3H, s, $-CH_3$)	2.23	0.05

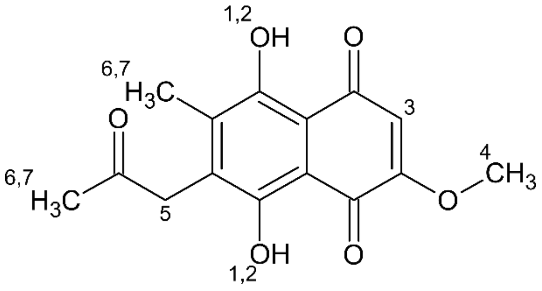

Supplement: Supplementary file 7 — Additional file 7. NMR table of javanicin isolated from Fs OE::fsr6. [file 40694_2019_89_MOESM7_ESM.pdf]
