# Supplementary material for: A new vector system for targeted integration and overexpression of genes in the crop pathogen Fusarium solani
Source: Fungal Biol Biotechnol. 2019 Dec 11;6:25. doi: 10.1186/s40694-019-0089-2 (PMC6905090; doi:10.1186/s40694-019-0089-2)
Supplement: Supplementary file 8 — Additional file 8. NMR table of bostrycoidin isolated from Fs OE::fsr6. [file 40694_2019_89_MOESM8_ESM.pdf]

Supplementary data for

"A new vector system for ectopic gene expression in the crop pathogen Fusarium solani"

by Nielsen MR, Holzwarth AKR, Brew E, Chrapkova N, Kaniki SEB, Kastaniegaard K, Sørensen T, Westphal KR,

Wimmer R, Sondergaard TE and Sørensen JL.

Additional file 8: NMR table of bostrycoidin isolated from *Fs* OE::*fsr6*

Signal	Bostrycoidin ppm (Yamamoto *et at*, 2002)[2]	ppm (obs)	ΔPPM
1	13.49 (1H, s, -OH)	13.46	0.03
2	13.20 (1H, s, -OH)	-	-
3	9.50 (1H, s, -CH)	9,47	0.03
4	7.96 (1H, s, -CH)	7.94	0.02
5	6.76 (1H, s, -CH)	6.74	0.02
6	4.02 (3H, s, $-CH_3$)	4.02	0.00
7	2.80 (3H, s, $-CH_3$)	2.80	0.00

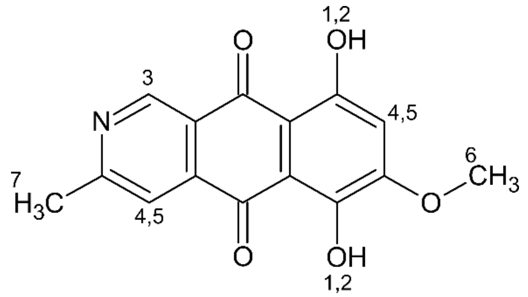

1 N. S. Chowdhury, M. H. Sohrab, M. S. Rana, C. M. Hasan, S. Jamshidi and K. M. Rahman, *J. Nat. Prod.*, 2017, **80**, 1173–1177.

2 Y. Yamamoto, Y. Kinoshita, G. Ran Thor, M. Hasumi, K. Kinoshita, K. Koyama, K. Takahashi and I. Yoshimura, *Phytochemistry*, 2002, **60**, 741–745.
